# Phytogenic Silver Nanoparticles Derived from *Ricinus communis* and *Aloe barbadensis*: Synthesis, Characterization, and Evaluation of Biomedical Potential

**DOI:** 10.3390/bioengineering12111273

**Published:** 2025-11-19

**Authors:** Anam Ahsan, George F. Gao, Wen-Xia Tian

**Affiliations:** 1 Centre for Pharmaceutical Innovation, University of South Australia, Adelaide, SA 5000, Australia; anam.ahsan@mymail.unisa.edu.au; 2 College of Animal Science & Veterinary Medicine, Shanxi Agricultural University, Taigu 030801, China; 3 Institute of Microbiology, Chinese Academy of Sciences, Shijingshan, Beijing 100043, China; gaof@im.ac.cn

**Keywords:** green synthesis, silver nanoparticles, *Ricinus communis*, *Aloe barbadensis*, biomedical applications

## Abstract

The green synthesis of silver nanoparticles (SNPs) using medicinal plants provides a sustainable and eco-friendly approach to nanoparticle production with promising biomedical potential. In this study, *Ricinus communis* and *Aloe barbadensis* aqueous leaf extracts were employed as reducing and stabilizing agents to synthesize *R. communis* SNPs (RcSNPs) and *A. barbadensis* SNPs (AbSNPs). The nanoparticles were characterized using ultraviolet–visible spectroscopy, dynamic light scattering, Fourier-transform infrared spectroscopy, scanning electron microscopy, transmission electron microscopy, thermogravimetric analysis, and differential scanning calorimetry to evaluate their physicochemical and thermal properties. RcSNPs and AbSNPs were predominantly spherical, with average sizes of 15–20 nm and 23–28 nm, respectively, and exhibited stability up to ~90 °C. Biological evaluations demonstrated potent antimicrobial, antioxidant, anti-inflammatory, anti-tyrosinase, and cytotoxic activities. Notably, RcSNPs and AbSNPs induced apoptosis through mitochondrial pathway modulation and showed superior cytotoxicity compared to crude plant extracts and several previously reported SNPs. These findings indicate that phytochemical-mediated SNPs not only provide a green route of synthesis but also exhibit multifunctional bioactivities, which may support their potential applications as antimicrobial, antioxidant, depigmenting, and anticancer agents in biomedical and pharmaceutical fields.

## 1. Introduction

Nanoscience has advanced rapidly, leading to the development of various nanoproducts that offer significant benefits to society [1]. Nanotechnology plays a crucial role in diverse fields such as biotherapy, pharmaceuticals, drug delivery, electronics, biotechnology, cosmetics, topical formulations (ointments and creams), nano-fertilizers, and water treatment [2]. Nanoparticles, defined as particulate matter with a diameter of less than 100 nm, exhibit unique properties that make them valuable in these applications [3,4]. Among these, silver nanoparticles (SNPs) have garnered widespread attention due to their exceptional electrical and thermal conductivity, as well as surface-enhanced Raman scattering [5,6,7]. Additionally, SNPs possess various biological activities, including antibacterial, antifungal, antioxidant, anti-inflammatory, and anticancer effects, and have potential use in gene therapy as non-viral carriers [8,9].

Several methods have been developed for the synthesis of SNPs, including chemical and electrochemical techniques. However, these approaches often encounter challenges during the purification stage due to the use of hazardous chemicals or the formation of toxic by-products, necessitating high energy input [10]. Moreover, controlling the size, shape, and achieving monodispersity of nanoparticles remain common challenges [10]. These limitations highlight the need for environmentally sustainable and safe synthesis methods. Consequently, green chemistry-based approaches have gained prominence in recent years [11,12].

Green synthesis involves the biological reduction of metal ions into nanoparticles using microorganisms or plant-derived extracts obtained from leaves, fruits, and seeds [13,14]. Plant extracts are rich in secondary metabolites such as alkaloids, terpenoids, flavonoids, enzymes, cyclic peptides, amino acids, proteins, polysaccharides, tannins, retinoic acid, ascorbic acid, polyphenols, and other compounds, which act as reducing agents, capping agents, and stabilizers during nanoparticle synthesis [15,16,17]. These compounds not only facilitate the synthesis process but also enhance the biological activity of the resulting nanoparticles. Furthermore, green synthesis offers advantages such as well-defined size and morphology, ease of scale-up, and high-yield production without surface contamination [12,18,19]. Recent reviews have highlighted that plant-mediated silver nanoparticles exhibit enhanced antimicrobial and cytotoxic activities through green synthesis routes [20,21,22].

Numerous plants and plant parts have been utilized for the biosynthesis of SNPs [14], including *Diospyros lotus* [11], *Zingiber officinale* [23], *Vernonia amygdalina* [24], *Sida rhombifolia* [25], grape seeds [26], *Physalis angulata* [27], *Camellia sinensis* [28], *Soymida febrifuga* [29], algae (*Parachlorella kessleri*) [30], *Mikania cordata* [31], chamomile (*Matricaria recutita*) [32], and *Ilex paraguariensis* [33]. The selection of plant sources often favors those that are readily available and renewable [34]. Factors such as extract concentration, metal salt concentration, temperature, pH, and reaction time significantly influence the rate of nanoparticle formation, yield, and physicochemical properties [12,35,36,37].

*Ricinus communis L.* (castor plant) and *Aloe barbadensis Mill.* (aloe vera) were selected for their rich phytochemical profiles and established therapeutic potential [38,39]. *R. communis* contains flavonoids, alkaloids, and phenolic acids known for antimicrobial and antioxidant properties [40], while *A. barbadensis* is rich in polysaccharides and phenolic compounds that facilitate metal ion reduction and stabilization [41]. These characteristics make both plants effective and sustainable candidates for green synthesis of metallic nanoparticles [42].

In this study, we aimed to synthesize optimized silver nanoparticles using the leaves of *Ricinus communis* L. and *Aloe barbadensis Mill*., perform detailed optimization of synthesis parameters, and evaluate their therapeutic potential.

## 2. Materials and Methods

### 2.1. Materials

Silver nitrate, bovine serum albumin (BSA), and other chemicals were purchased from Sigma-Aldrich (St. Louis, MO, USA). B16F10 murine melanoma and HepG2 human hepatocarcinoma cell lines were obtained from the Cell Bank of the Chinese Academy of Sciences (Shanghai, China). RPMI-1640, Dulbecco’s Modified Eagle’s Medium (DMEM), fetal bovine serum (FBS), and trypsin were supplied by Thermo Fisher Scientific (Waltham, MA, USA). All other reagents were of analytical grade, and double-distilled water was used throughout.

### 2.2. Methods

#### 2.2.1. Preparation of Plant Extracts

Fresh leaves of *Ricinus communis L.* and *Aloe barbadensis Mill.* were collected from Shanxi Agricultural University, washed with running and double-distilled water, and air-dried for 10–12 days at room temperature. Approximately 20 g of powdered *R. communis* leaves and sliced *A. barbadensis* leaves were separately boiled in 200 mL distilled water for 30 min. The extracts were cooled, filtered through Whatman No. 1 paper, and stored at 4 °C until further use. For methanolic extracts, 10 g of dried leaf powder was soaked in 100 mL methanol for 48 h, filtered, and concentrated under reduced pressure.

Distilled water was used to maintain a fully green synthesis protocol, as hydrophilic phytochemicals (phenolics, flavonoids, glycosides) are sufficient to reduce Ag^+^ ions and stabilize nanoparticles. This approach aligns with green chemistry principles and has been validated in multiple plant-based SNP syntheses [43].

#### 2.2.2. Phytochemical Analysis

Qualitative screening followed established phytochemical tests as described by Trease and Evans [44], Harborne [45], and Sofowora [46], including Dragendorff’s for alkaloids, ferric chloride for phenolics, foam test for saponins, and Liebermann–Burchard for steroids [47]. Qualitative screening of aqueous leaf extracts was performed to detect alkaloids, phenols, proteins, carbohydrates, glycosides, steroids, saponins, tannins, flavonoids, and terpenoids using standard procedures [48,49,50].

#### 2.2.3. Total Phenolic Content (TPC) Estimation

TPC was determined using a modified Folin–Ciocalteu method [51]. Briefly, 100 µg/mL of plant extract or gallic acid standard was mixed with 0.5 mL Folin–Ciocalteu reagent, incubated in the dark for 10 min, followed by addition of 2.5 mL 20% sodium carbonate. After 1 h in the dark, absorbance was measured at 765 nm using a UV–Vis spectrophotometer (UV-2600i, Shimadzu, Kyoto, Japan).

#### 2.2.4. Biogenic Synthesis of Silver Nanoparticles (SNPs)

A 1 mM AgNO_3_ solution (100 mL) was prepared. For *R. communis* silver nanoparticle (RcSNP) or *A. barbadensis* silver nanoparticle (AbSNP) synthesis, 1 mL of *R. communis* or *A. barbadensis* leaf extract was added dropwise to 9 mL AgNO_3_ in separate flasks. The mixtures were shaken, and incubated in the dark at room temperature, and a control without extract was maintained. SNP solutions were purified by repeated centrifugation (10,000 rpm, 15 min) and redispersed in deionized water three times, and then freeze-dried for further characterization.

#### 2.2.5. Optimization of SNP Synthesis

AgNO_3_ concentrations (0.05–4 mM) and extract volumes (1–4 mL) were varied to optimize SNP synthesis. With optimized concentrations, reaction time (30 min–24 h), temperature (25–110 °C), and pH (2–13) were systematically evaluated. UV–Vis spectra were recorded at 300–800 nm, and all glassware was covered to prevent photo-degradation [52,53].

#### 2.2.6. UV–Visible Spectroscopy

Absorption spectra of SNPs were recorded using a Shimadzu UV-2600i spectrophotometer (UV-2600i, Shimadzu, Kyoto, Japan) at 300–800 nm with 1 mL aliquots of diluted reaction mixtures at various time intervals.

#### 2.2.7. Stability Study

Purified SNPs were stored in the dark at ambient conditions. Stability was monitored via UV–Vis spectroscopy (300–800 nm) over a period of up to 30 days.

#### 2.2.8. Characterization of SNPs

**DLS and Zeta Potential:** Hydrodynamic size and surface charge were measured using a ZetaPALS dynamic light scattering instrument (Brookhaven Instruments Corporation, Holtsville, NY, USA). SNPs (5 mg) were dispersed in 5 mL water, sonicated, and measured at 689 nm, 90 °C, with three readings per sample.

**FTIR Spectroscopy:** FTIR spectra of leaf extracts and SNPs were obtained using KBr pellets on a Fourier Transform Infrared Spectrophotometer (IRAffinity-1, Shimadzu, Kyoto, Japan) in the 400–4000 cm^−1^ range with 4 cm^−1^ resolution.

**SEM Analysis:** Morphology was visualized using a Scanning Electron Microscope (JSM-5600, JEOL Ltd., Tokyo, Japan) on thin films of SNPs prepared on carbon-coated copper grids.

**TEM Analysis:** Particle size and morphology were confirmed using a Transmission Electron Microscope (JEM-100CX II, JEOL Ltd., Tokyo, Japan) with SNP suspensions (1 µg/mL) placed on copper grids dried at 37 °C.

**TGA and DSC:** Thermal stability was assessed using a Thermogravimetric/Differential Scanning Calorimetry Analyzer (STA 7300, Hitachi High-Tech, Tokyo, Japan). Lyophilized SNPs (8.6 mg) were heated from 40 °C to 900 °C at 10 °C/min under N_2_ flow (40 mL/min), with reference crucibles used for comparison.

#### 2.2.9. Antimicrobial Activity

The antimicrobial potential of RcSNPs and AbSNPs was assessed using the agar well diffusion method, which allows direct application of nanoparticle suspensions [54]. Bacterial strains (*Staphylococcus aureus* ATCC 25923, *Escherichia coli* ATCC 25922, *Salmonella typhi* ATCC 6539) and fungal strains (*Candida albicans* ATCC 10231, *Aspergillus niger* ATCC 16404) were obtained from the Microbial Culture Collection, Shanxi Agricultural University (Taigu, China). Gentamicin sulfate (≥98%) and fluconazole (≥99%) were purchased from Sigma-Aldrich (St. Louis, MO, USA) and served as positive controls.

**Agar Well Diffusion:** Cultures were standardized to 0.5 McFarland (~1.5 × 10^8^ CFU/mL). Mueller–Hinton agar (for bacteria) or potato dextrose agar (for fungi) plates were obtained from Oxoid Ltd. (Basingstoke, UK).SNP solutions (50 μg/mL, 50–100 μL per well) were added to wells andplates were incubated at 37 °C for 24 h, and zones of inhibition were measured [55].

#### 2.2.10. In Vitro Anti-Inflammatory Assays

**Protein Denaturation:** 500 μL of bovine serum albumin (BSA, ≥96%, Sigma-Aldrich, St. Louis, MO, USA) (1%) was mixed with 100 μL extract or SNPs (pH 6.3), incubated at 37 °C for 20 min, then heated at 55 °C for 20 min. Absorbance at 660 nm was measured using a UV–Vis spectrophotometer (UV-2600i, Shimadzu, Kyoto, Japan). Acetylsalicylic acid (aspirin, ≥99%, Sigma-Aldrich, St. Louis, MO, USA) was used as a positive control [56]. The following equation estimated the inhibition (%) of protein denaturation:

(1) % Inhibition = (A_control − A_sample) / A_control × 100

where:

A_control_ = absorbance of the control sample
A_sample_ = absorbance of the test sample

**Protease Inhibition:** 100 μL extract/SNPs was mixed with 100 μL BSA and incubated for 5 min; then, 250 μL of trypsin (from porcine pancreas, ≥10,000 BAEE units/mg, Sigma-Aldrich, St. Louis, MO, USA) was added. Supernatant absorbance at 210 nm was taken to measure protease inhibition [57].

#### 2.2.11. In Vitro Antioxidant Assays

**DPPH Assay:** 250 μL of 0.3 mM 2,2-diphenyl-1-picrylhydrazyl (DPPH, Sigma-Aldrich, St. Louis, MO, USA) solution was mixed with 2.5 mL extract/SNPs (10–100 μg/mL). Butylated hydroxytoluene (BHT, ≥99%, Sigma-Aldrich, St. Louis, MO, USA) served as a positive control, and absorbance was recorded at 517 nm [58].

**H_2_O_2_ Scavenging:** Extracts/SNPs (10–100 μg/mL) were mixed with 50 μL of 5 mM hydrogen peroxide (H_2_O_2_, Merck, Darmstadt, Germany), incubated for 20 min, and the absorbance was recorded at 610 nm [59].

**Nitric Oxide Scavenging:** Sodium nitroprusside (≥98%, Sigma-Aldrich, St. Louis, MO, USA)was used to generate nitric oxoid (NO), and incubated with extract/SNPs (10–100 μg/mL) for 60 min; nitrite was measured using Griess reagent [60].

**Reducing Power:** Extracts/SNPs (10–100 μg/mL) were mixed with phosphate buffer (0.2 M, pH 6.6) and 1% potassium ferricyanide (K_3_[Fe(CN)_6_], Merck, Darmstadt, Germany) incubated at 50 °C for 20 min, cooled, and mixed with 10% trichloroacetic acid (TCA, Sigma-Aldrich, St. Louis, MO, USA). The mixture was centrifuged at 3000 rpm for 10 min; the supernatant was combined with 0.1% ferric chloride (FeCl_3_, Merck, Darmstadt, Germany), and absorbance was measured at 700 nm [61].

#### 2.2.12. Tyrosinase Inhibition

SNPs were tested with L-tyrosine (≥98%, Sigma-Aldrich, St. Louis, MO, USA) and mushroom tyrosinase (≥1000 units/mg solid, Sigma-Aldrich, St. Louis, MO, USA) in 50 mM potassium phosphate buffer (pH 6.8, Thermo Fisher Scientific, Waltham, MA, USA) at 30 °C. The reaction mixture was pre-incubated for 10 min, and absorbance at 492 nm was recorded using a microplate reader (Multiskan Sky, Thermo Fisher Scientific, Waltham, MA, USA). Percentage inhibition was calculated accordingly [62].

#### 2.2.13. In Vitro Cytotoxicity

**Cell Culture:** B16F10 (ATCC CRL-6475) and HepG2 (ATCC HB-8065) cell lines were obtained from the Cell Bank of the Chinese Academy of Sciences (Shanghai, China) and used at passages 10–15. B16F10 and HepG2 cells were maintained at 37 °C, 5% CO_2_, detached with 0.25% trypsin, and seeded at 3 × 10^3^ cells/well in 96-well plates.

**Cytotoxicity Evaluation:** Cells treated with SNPs (0–100 μg/mL) for 24 h. MTT assay was performed using 3-(4,5-dimethylthiazol-2-yl)-2,5-diphenyltetrazolium bromide (MTT, Sigma-Aldrich, St. Louis, MO, USA), and formazan crystals were dissolved in dimethyl sulfoxide (DMSO, Sigma-Aldrich, St. Louis, MO, USA). Absorbance was recorded at 570 nm using a microplate reader (Multiskan Sky, Thermo Fisher Scientific, Waltham, MA, USA) [63].

Cell viability (%) = (OD _sample_/OD _control_) × 100 (2)

where

OD_sample_ = absorbance of treated cells
OD_control_ = absorbance of untreated cells used as control.

#### 2.2.14. Statistical Analysis

All experiments were performed in triplicate. Data are presented as mean ± SD and were analyzed using one-way ANOVA with GraphPad Prism v5 (GraphPad Software Inc., San Diego, CA, USA).

## 3. Results

### 3.1. Phytochemical Analysis

Phytochemical screening of aqueous and methanol leaf extracts of *Ricinus communis and Aloe barbadensis* revealed the presence of various bioactive constituents. The results, summarized in Table 1, indicate that both plant extracts contain key biomolecules such as alkaloids, phenols, proteins, carbohydrates, glycosides, steroids, saponins, tannins, flavonoids, and terpenoids, which may contribute to their therapeutic potential.

### 3.2. Visual Inspection

**Figure 1:**
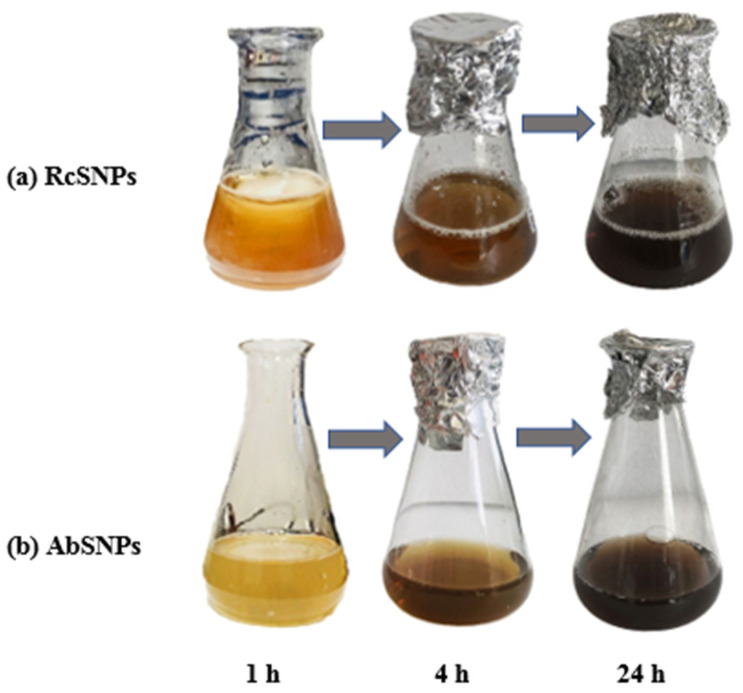
Color change in the reaction mixture of silver nanoparticles synthesized from *Ricinus communis* (RcSNPs, (**a**)) and *Aloe barbadensis* (AbSNPs, (**b**)) over time (1 h, 4 h, and 24 h).

The synthesis of silver nanoparticles (SNPs) can be initially indicated by a distinct color change in the reaction mixture, which occurs due to the excitation of surface plasmon resonance (SPR) of SNPs [14,64]. Typically, the solution transitions from colorless to yellow, pale yellow, yellowish-brown, and eventually to dark brown. In the present study, a progressive change in color from yellowish to light brown, and finally to dark brown, was observed upon addition of the plant extract to silver nitrate solution, confirming the formation of SNPs (Figure 1). The synthesis method used has been previously shown to be reproducible; in our experiments, the nanoparticle properties were consistent with prior reports [65,66]. This consistency confirms the reliability and stability of the adopted phytogenic synthesis approach.

### 3.3. UV-Visible Spectroscopy

UV–visible (UV–vis) spectroscopy is a simple and widely used technique for monitoring the synthesis of silver nanoparticles (SNPs) [67]. In this study, reactions were carried out at room temperature. The characteristic surface plasmon resonance (SPR) bands observed near 435 nm for *R. communis* and 442 nm for *A. barbadensis* confirmed the reduction of Ag^+^ ions to metallic silver. Although water absorption bands are predominant in KBr FTIR spectra, characteristic peaks corresponding to hydroxyl (~3400 cm^−1^), carbonyl (~1635 cm^−1^), and amine (~1385 cm^−1^) groups were consistent with functional groups reported for plant extracts and confirm biomolecule involvement in Ag^+^ reduction and nanoparticle stabilization [68].

To optimize the synthesis, key parameters including AgNO_3_ concentration, extract concentration, reaction time, temperature, and pH were systematically varied, as these factors strongly influence nanoparticle formation [36].

#### 3.3.1. Effect of AgNO_3_ Concentration

**Figure 2:**
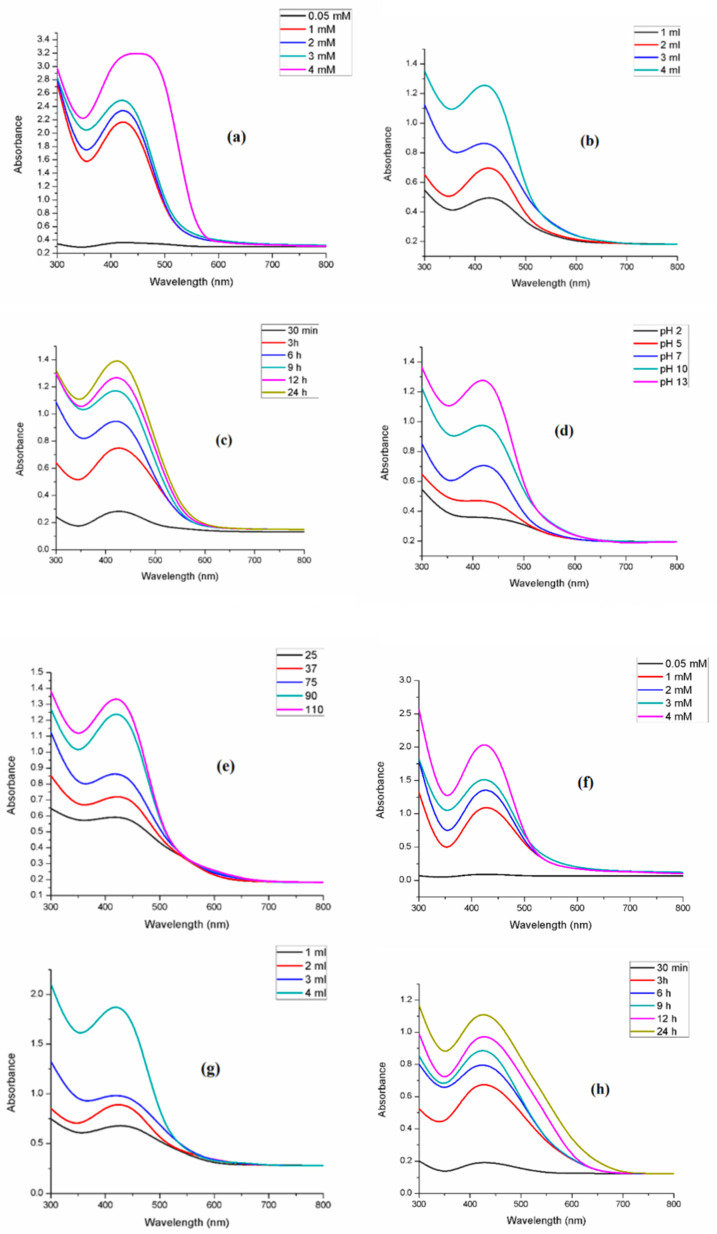

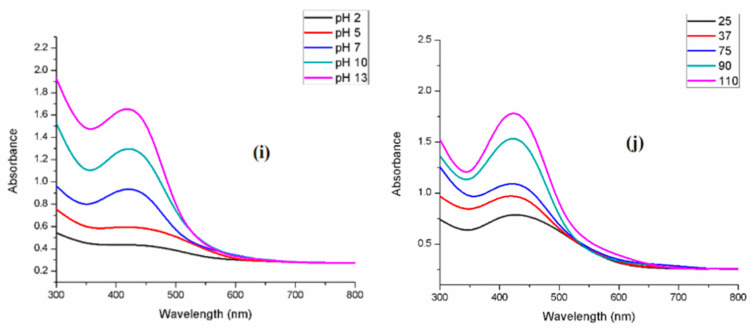
UV–visible spectra of silver nanoparticles synthesized from *Ricinus communis* (RcSNPs) and *Aloe barbadensis* (AbSNPs) showing the effects of varying parameters: AgNO_3_ concentration (**a**,**f**), leaf extract concentration (**b**,**g**), reaction time (**c**,**h**), pH (**d**,**i**), and temperature (**e**,**j**). Data represent mean ± SD (*n* = 3).

SNPs were synthesized using varying concentrations of AgNO_3_ (0.5–4 mM), while maintaining the extract concentration at 1 mL under constant reaction conditions. No SPR band was detected at low AgNO_3_ concentrations; however, distinct peaks were observed at 435 nm for 1 mM AgNO_3_ with *R. communis* extract, and at 442 nm for 2 mM AgNO_3_ with *A. barbadensis* extract. Increasing AgNO_3_ concentration led to higher peak intensity and peak broadening, indicating an increase in SNP concentration and particle aggregation (Figure 2a,f) [40]. Based on these results, the optimal AgNO_3_ concentrations were set at 1 mM for *R. communis* and 2 mM for *A. barbadensis* for subsequent experiments.

#### 3.3.2. Effect of Leaf Extract Concentration

Extract concentration also influenced nanoparticle properties. Optimization was performed by fixing the AgNO_3_ concentration and varying the extract volume (1–4 mL). With increasing extract concentration, the SPR bands became narrower, suggesting improved monodispersity of SNPs (Figure 2b,g). An extract volume of 1 mL was determined to be optimal for further experiments.

#### 3.3.3. Effect of Reaction Time

Reaction time significantly affected SNP formation and stability. At the initial stage, no SPR peak appeared immediately after mixing silver nitrate with plant extracts. After 1 h, characteristic SPR bands at 435 nm (*R. communis*) and 442 nm (*A. barbadensis*) emerged, with peak intensity increasing over time. Maximum absorbance was recorded at 6 h for *R. communis* and 9 h for *A. barbadensis* (Figure 2c,h). Beyond these times, peak broadening occurred, likely due to agglomeration or increased particle size. Therefore, 6 h and 9 h were selected as the optimized reaction times for *R. communis* and *A. barbadensis*, respectively.

#### 3.3.4. Effect of pH

pH had a notable influence on the morphology and stability of SNPs. Higher pH values enhanced the reduction of Ag^+^ ions, resulting in stronger absorbance and improved stability of the nanoparticles (Figure 2d,i).

#### 3.3.5. Effect of Temperature

Temperature variations also affected SNP synthesis. Although different temperatures were tested, room temperature was found to be optimal, producing small, spherical SNPs with a sharp and distinct single SPR band at shorter wavelengths (Figure 2e,j).

### 3.4. Stability of Synthesized SNPs

**Figure 3:**
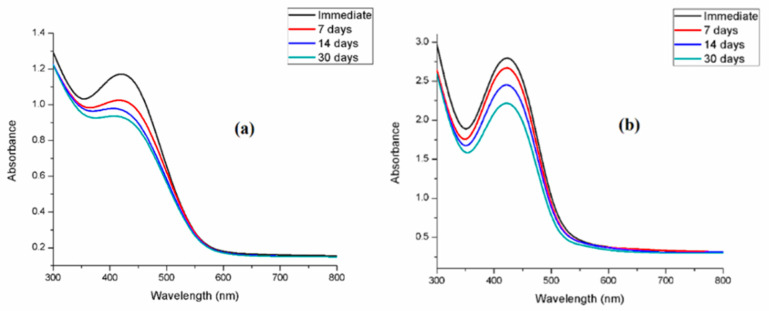
Stability studies of silver nanoparticles synthesized from *Ricinus communis* (RcSNPs (**a**)) and *Aloe barbadensis* (AbSNPs (**b**)) over a one-month period. Data represent mean ± SD (*n* = 3).

The stability of biosynthesized SNPs was evaluated by monitoring their UV–vis spectra over time. As shown in Figure 3a,b, prolonged storage led to a gradual decrease in absorbance intensity and band broadening, indicating possible agglomeration or partial release of metallic ions. Importantly, the SPR band position remained unchanged even after 30 days, suggesting that the nanoparticles retained their structural integrity [69]. In addition, reproducibility was confirmed by synthesizing SNPs in triplicate over a period of six months. The spectra remained consistent, demonstrating that *R. communis* and *A. barbadensis* extract-mediated SNPs possess excellent stability and dispersity.

### 3.5. Dynamic Light Scattering (DLS) and Zeta Potential (ZP) Analysis

**Figure 4:**
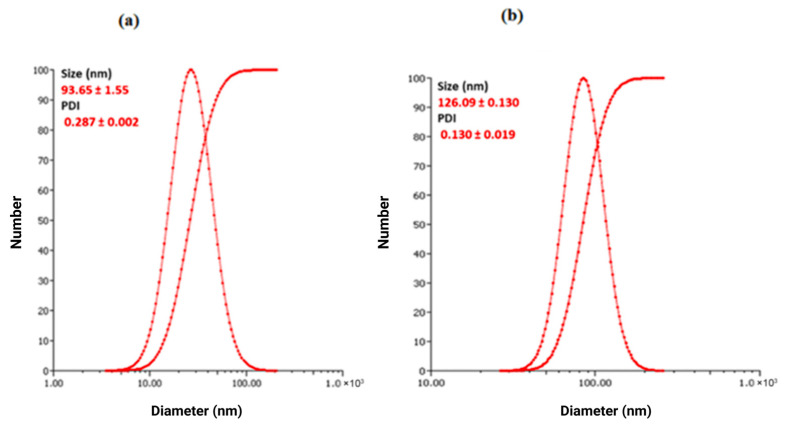
Dynamic light scattering (DLS) size-distribution histograms of silver nanoparticles synthesized from *Ricinus communis* (RcSNPs (**a**)) and *Aloe barbadensis* (AbSNPs (**b**)). The X-axis represents particle diameter (nm) and the Y-axis represents number distribution (%). The average hydrodynamic diameters were 93.7 ± 1.6 nm (RcSNPs) and 126.1 ± 0.1 nm (AbSNPs), with polydispersity indices (PDI) of 0.29 ± 0.002 and 0.13 ± 0.02, respectively (*n* = 3).

Dynamic light scattering (DLS) is a widely used technique for determining nanoparticle size distribution at the nanometer scale [70]. The DLS profiles of biogenic SNPs are presented in Figure 4a,b. Under optimized conditions, the average hydrodynamic diameter of RcSNPs and AbSNPs was 93.65 ± 1.55 nm and 126.09 ± 0.13 nm, respectively. The DLS histograms (Figure 4) show narrow and unimodal size distributions, confirming the uniformity of the RcSNP and AbSNP populations. The corresponding polydispersity index (PDI) values were 0.287 ± 0.002 and 0.130 ± 0.019, indicating narrow size distributions. The zeta potential (ZP) values were −67.09 mV (RcSNPs) and −83.42 mV (AbSNPs), confirming strong surface charge and excellent colloidal stability. These findings are consistent with the UV–vis analysis results.

### 3.6. Fourier Transform Infrared (FTIR) Spectroscopy

Fourier transform infrared (FTIR) spectroscopy was employed to investigate the surface chemistry of SNPs and to identify biomolecules responsible for reducing Ag^+^ ions and stabilizing the nanoparticles [71]. The FTIR spectra of both plant extracts and their corresponding SNPs were compared to identify characteristic peaks and spectral shifts.

**Figure 5:**
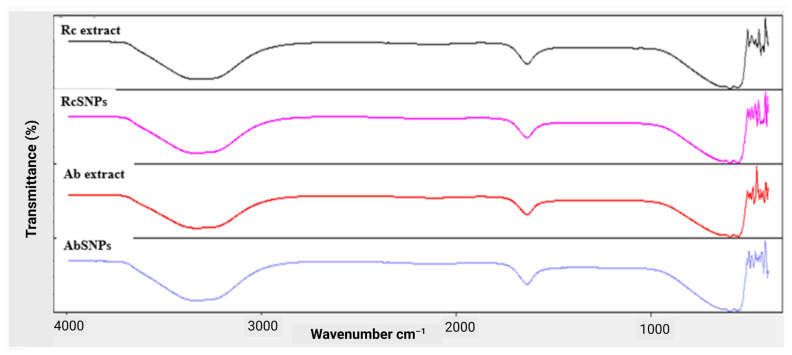
Fourier transform infrared (FTIR) spectra of *Ricinus communis* and *Aloe barbadensis* leaf extracts, and their corresponding silver nanoparticles (RcSNPs and AbSNPs).

For RcSNPs, prominent absorption bands were observed at 3326 cm^−1^ and 1720 cm^−1^, while AbSNPs exhibited bands at 3576 cm^−1^ and 1850 cm^−1^ (Figure 5). The bands at 3326 cm^−1^ (RcSNPs) and 3576 cm^−1^ (AbSNPs) correspond to –OH stretching vibrations, whereas the bands at 1720 cm^−1^ and 1850 cm^−1^ are attributed to N–H stretching of amine groups, indicating the capping of SNPs by these functional groups. Additionally, lower intensity bands at 2105 cm^−1^ (RcSNPs) and 2157 cm^−1^ (AbSNPs) were detected, corresponding to carbonyl (C = O) stretching. Although the FTIR spectrum is dominated by water peaks, the presence of functional groups such as –OH, C = O, and –NH was inferred from comparative analysis with literature reports [68,72].

### 3.7. Scanning Electron Microscopy (SEM) Analysis

**Figure 6:**
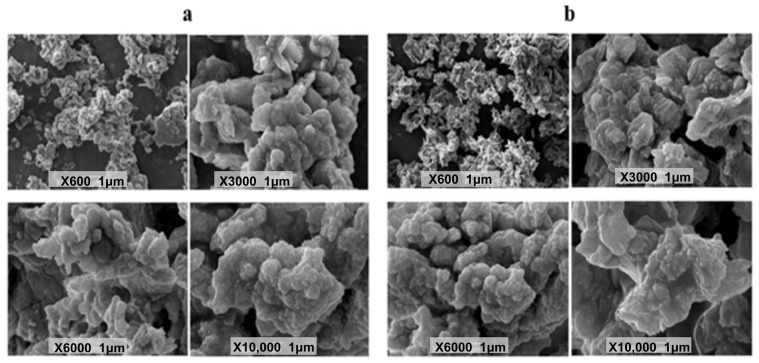
Scanning electron microscopy (SEM) images of silver nanoparticles synthesized from *Ricinus communis* (RcSNPs (**a**)) and *Aloe barbadensis* (AbSNPs (**b**)) at different magnifications (×600, ×3000, ×6000, ×10,000).

Scanning electron microscopy (SEM) was used to examine the morphology and size of the biosynthesized SNPs. The micrographs revealed nanoparticles with spherical, cubic, triangular, and rectangular shapes, distributed relatively evenly. Some degree of agglomeration was observed, likely due to the limited stabilization provided by weak capping agents [73]. The agglomerated particle sizes ranged from 256 to 272 nm for RcSNPs and 263 to 278 nm for AbSNPs, while the average particle sizes were estimated to be 68 nm (RcSNPs) and 74 nm (AbSNPs) (Figure 6a,b).

### 3.8. Transmission Electron Microscopy (TEM) Analysis

**Figure 7:**
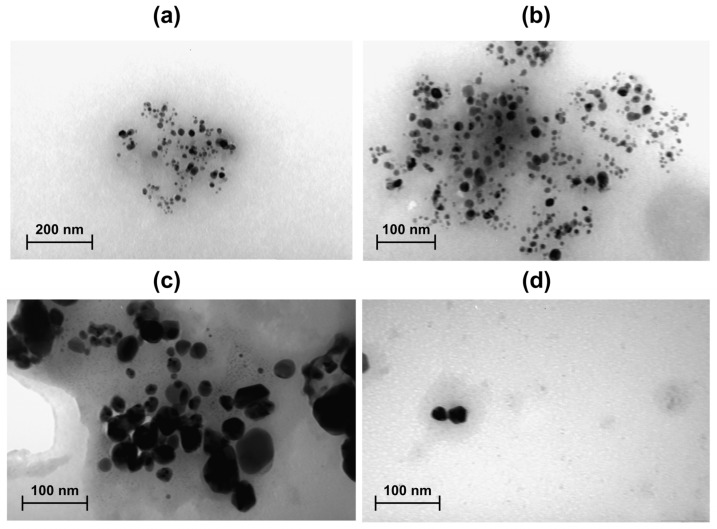
Transmission electron microscopy (TEM) images of silver nanoparticles synthesized from *Ricinus communis* (RcSNPs (**a**,**b**)) and *Aloe barbadensis* (AbSNPs (**c**,**d**)) at different magnifications.

Transmission electron microscopy (TEM) provided further insights into the morphology and size of the biosynthesized SNPs (Figure 7a–d). The images revealed that most nanoparticles were spherical, consistent with the observations from UV–vis and SEM analyses. Particle size measurements performed using ImageJ software (version 1.52a) indicated sizes of 15–20 nm for RcSNPs and 23–28 nm for AbSNPs.

### 3.9. Thermogravimetric Analysis (TGA) and Differential Scanning Calorimetry (DSC)

**Figure 8:**
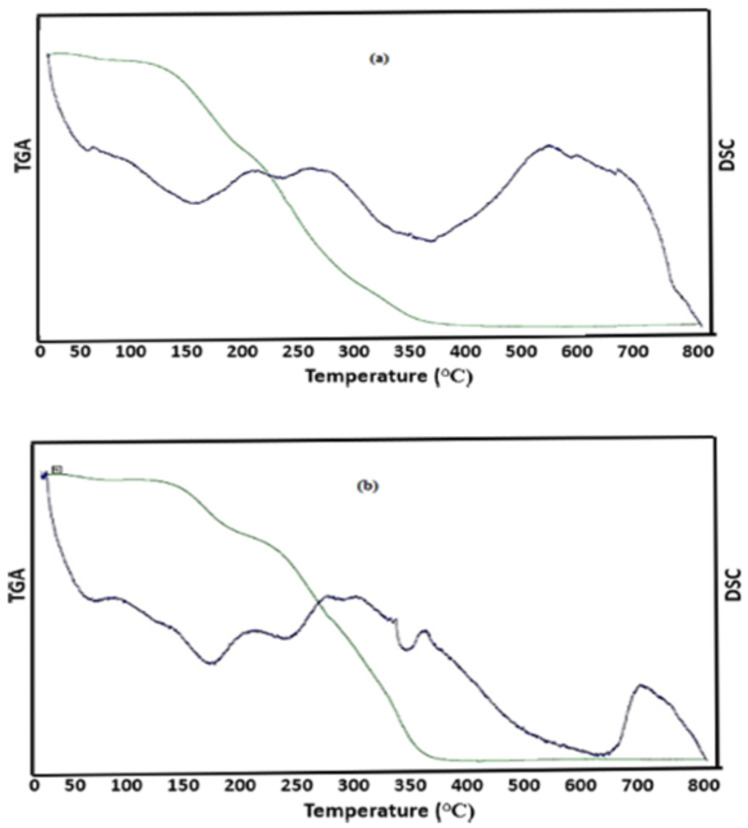
Thermogravimetric (TGA) and differential scanning calorimetry (DSC) thermograms of silver nanoparticles synthesized from *Ricinus communis* (RcSNPs, (**a**) and *Aloe barbadensis* (AbSNPs, (**b**). Green line represents TGA while blue line represents DSC.

Thermogravimetric analysis (TGA) was performed to evaluate the thermal stability of the biosynthesized SNPs. The results indicated that RcSNPs began to degrade at approximately 200 °C, while AbSNPs showed initial degradation at 240 °C. A gradual weight loss was observed up to 800 °C (Figure 8a,b). Differential scanning calorimetry (DSC) revealed endothermic peaks at 190 °C for RcSNPs and 220 °C for AbSNPs, corresponding to thermal transitions of the nanoparticles (Figure 8a,b).

### 3.10. Antimicrobial Activity of Biogenic SNPs

**Figure 9:**
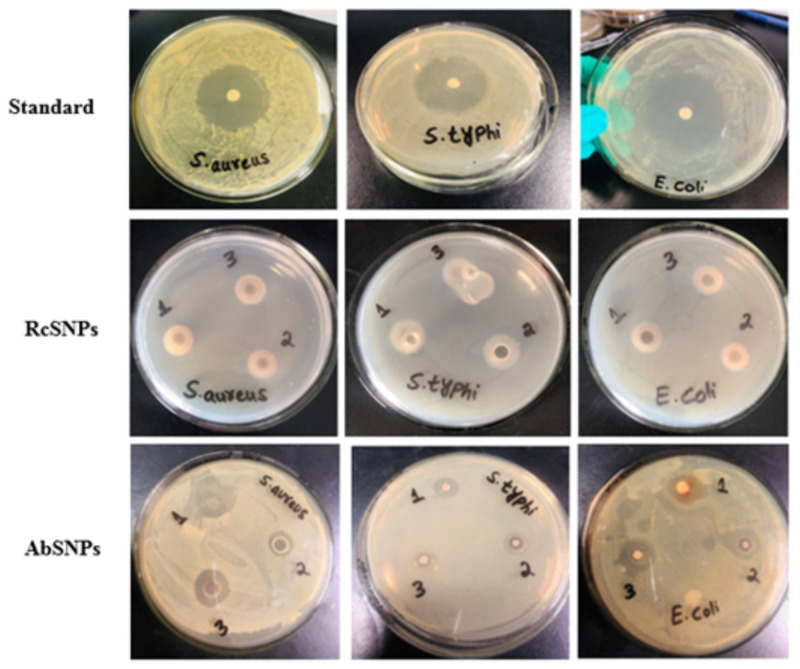
Antibacterial activity of silver nanoparticles synthesized from *Ricinus communis* (RcSNPs) and *Aloe barbadensis* (AbSNPs) against pathogenic bacterial strains. Treatments: leaf extract (1), 50 µL SNPs (2), 100 µL SNPs (3). Data represent mean ± SD (*n* = 3).

**Figure 10:**
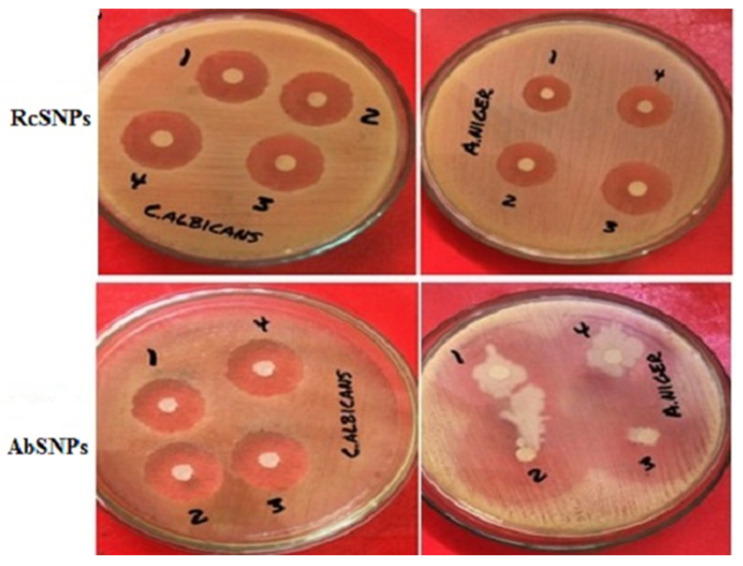
Antifungal activity of silver nanoparticles synthesized from *Ricinus communis* (RcSNPs) and *Aloe barbadensis* (AbSNPs) against pathogenic fungal strains. Treatments: leaf extract (1), fluconazole (2), 50 µL SNPs (3), 100 µL SNPs (4). Data represent mean ± SD *(n* = 3).

The antimicrobial potential of RcSNPs and AbSNPs was evaluated against selected bacterial and fungal pathogens, and their efficacy was quantified by measuring the zones of inhibition (mm) (Figure 9 and Figure 10; Table 2).

RcSNPs exhibited zones of inhibition of 22.3 ± 0.8 mm against *C. albicans* and 20.2 ± 0.9 mm against *S. aureus*, followed by 18.4 ± 0.7 mm, 19.3 ± 0.5 mm, and 21.4 ± 0.7 mm against *A. niger*, *S. typhi*, and *E. coli*, respectively. For AbSNPs, the corresponding zones were 21.8 ± 0.4 mm and 20.3 ± 0.05 mm against *A. niger* and *S. aureus*, and 24.3 ± 0.5 mm, 18.6 ± 0.07 mm, and 17.3 ± 0.05 mm against *C. albicans*, *S. typhi*, and *E. coli*, respectively (Figure 9 and Figure 10).

Both SNPs demonstrated the highest antibacterial activity against *S. aureus*, followed by *S. typhi* and *E. coli*. When compared to the aqueous leaf extracts of *R. communis* and *A. barbadensis*, RcSNPs showed enhanced antibacterial activity against *S. typhi*, whereas AbSNPs were more effective against *E. coli*. Regarding antifungal activity, RcSNPs exhibited superior inhibition of *C. albicans*, while AbSNPs were more active against *A. niger*. Notably, the antifungal activity of both SNPs exceeded that of the standard antifungal agent, fluconazole, highlighting their strong potential as antimicrobial agents.

### 3.11. In Vitro Anti-Inflammatory Assay

**Figure 11:**
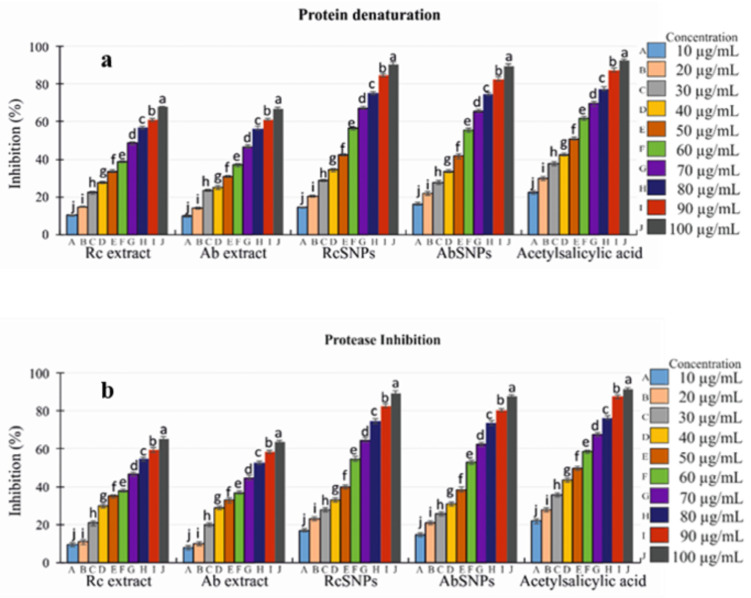
In vitro anti-inflammatory activity of *Ricinus communis* (Rc) and *Aloe barbadensis* (Ab) extracts and their corresponding SNPs. (**a**) Heat-induced protein denaturation; (**b**) Protease inhibition. Values with different superscripts (a–j) within the same variable are significantly different (*p* < 0.05).

The anti-inflammatory potential of *R. communis* and *A. barbadensis* extracts and their corresponding SNPs was evaluated using the heat-induced albumin denaturation method. The plant extracts exhibited 67.45 ± 1.7% (*R. communis*) and 66.03 ± 1.8% (*A. barbadensis*) inhibition, whereas RcSNPs and AbSNPs showed significantly higher activity at 90.67 ± 1.7% and 89.79 ± 1.7%, respectively. Acetylsalicylic acid was used as the standard anti-inflammatory agent (Figure 11a). A concentration-dependent increase in inhibition was observed for both extracts and SNPs.

Furthermore, the extracts and SNPs demonstrated notable antiprotease activity. The standard drug, acetylsalicylic acid, exhibited the highest protease inhibitory effect (95.37 ± 1.3%). R. communis and *A. barbadensis* extracts showed 65.45 ± 1.6% and 63.03 ± 1.7% inhibition, respectively, whereas RcSNPs and AbSNPs displayed enhanced activity at 89.67 ± 1.4% and 87.79 ± 1.3%, respectively (Figure 11b).

### 3.12. In Vitro Antioxidant Assay

Free radicals are implicated in numerous human diseases, including arthritis, atherosclerosis, reperfusion injury, ischemia, central nervous system damage, gastritis, AIDS, and cancer. Recently, natural antioxidants have gained considerable attention due to their potential health benefits [73]. The antioxidant potential of *Ricinus communis* (Rc) and *Aloe barbadensis* (Ab) extracts and their corresponding SNPs was evaluated using DPPH, hydrogen peroxide (H_2_O_2_), nitric oxide (NO) scavenging, and reducing power assays.

**Figure 12:**
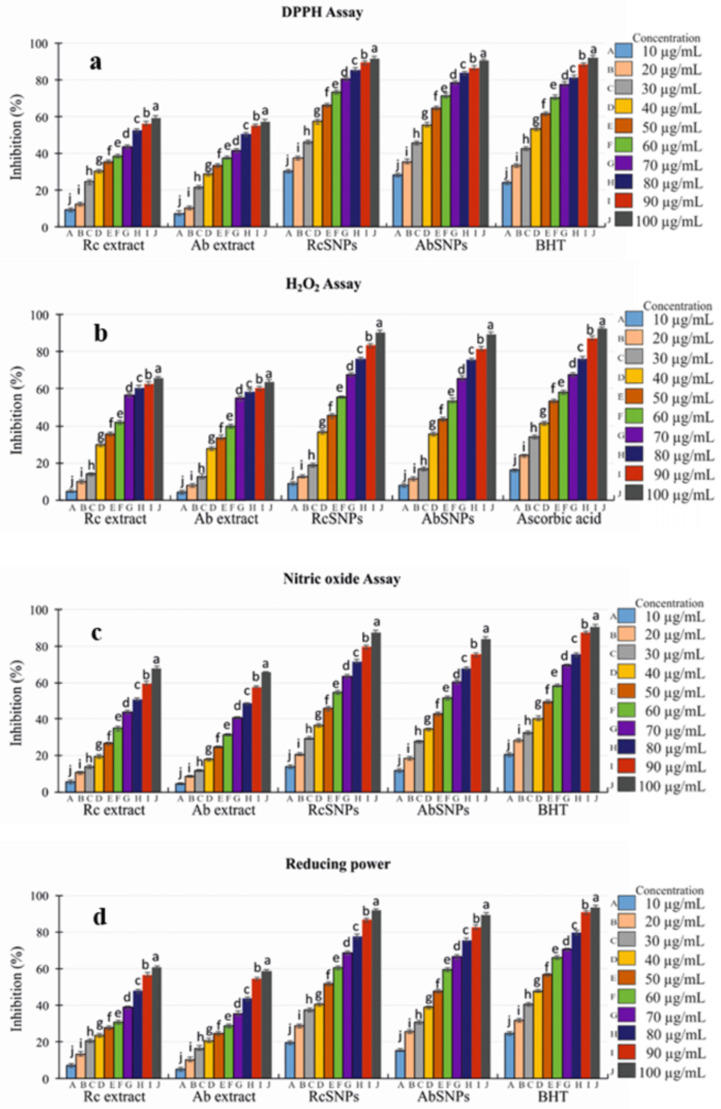
In vitro antioxidant activity of *Ricinus communis* (Rc) and *Aloe barbadensis* (Ab) extracts and their corresponding SNPs. (**a**) DPPH assay; (**b**) H_2_O_2_ scavenging assay; (**c**) NO scavenging assay; (**d**) Reducing power assay. Values with different superscripts (a–j) within the same variable are significantly different (*p* < 0.05).

In the DPPH assay, RcSNPs and AbSNPs demonstrated significant, concentration-dependent radical scavenging activity, with increasing inhibition observed at higher concentrations (10–100 µg/mL) (Figure 12a). Similarly, H_2_O_2_ scavenging activity was assessed spectrophotometrically, and RcSNPs, AbSNPs, and ascorbic acid exhibited 90.57 ± 0.9%, 89.02 ± 0.7%, and 92.05 ± 0.4% inhibition at 100 µg/mL, respectively (Figure 12b). H_2_O_2_ scavenging activity was slightly lower than DPPH scavenging for both SNPs, suggesting differential efficacy against specific reactive species.

Biogenic RcSNPs and AbSNPs also exhibited NO scavenging activity in a dose-dependent manner, achieving 87.41 ± 1.8% and 83.87 ± 1.7% inhibition at 100 µg/mL, respectively, slightly lower than the standard BHT (90.83 ± 1.8%) (Figure 12c). The reducing power of the SNPs similarly increased with concentration, with maximum activities of 91.41 ± 1.6% (RcSNPs) and 89.08 ± 1.7% (AbSNPs), comparable to BHT (93.76 ± 1.7%) (Figure 12d).

Overall, these results indicate that both RcSNPs and AbSNPs possess strong, concentration-dependent antioxidant potential, comparable to standard antioxidant agents, highlighting their promising therapeutic value.

### 3.13. Tyrosinase Inhibition Activity

RcSNPs and AbSNPs exhibited potent tyrosinase inhibitory activity, with inhibition percentages of 97.98 ± 1.5% and 98.43 ± 1.5%, respectively (Table 3). These results indicate that both biogenic SNPs are highly effective in inhibiting tyrosinase, suggesting their potential applications in skin-whitening and anti-melanogenic formulations.

### 3.14. In Vitro Cytotoxicity Evaluation

Given the diverse biological applications of silver nanoparticles, the anticancer potential of RcSNPs and AbSNPs was evaluated using the MTT assay [74,75]. B16F10 and HepG2 cells were treated with varying concentrations of biogenic RcSNPs and AbSNPs (0–100 µg/mL) for 24 and 48 h.

**Figure 13:**
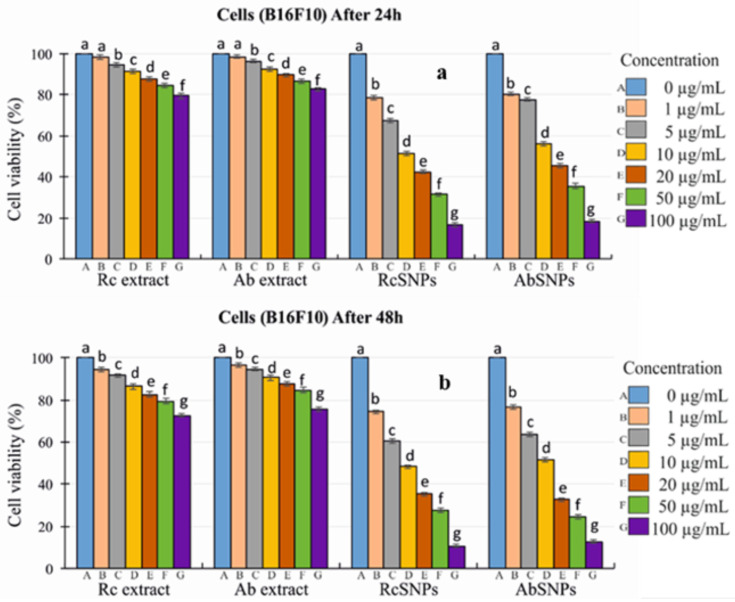

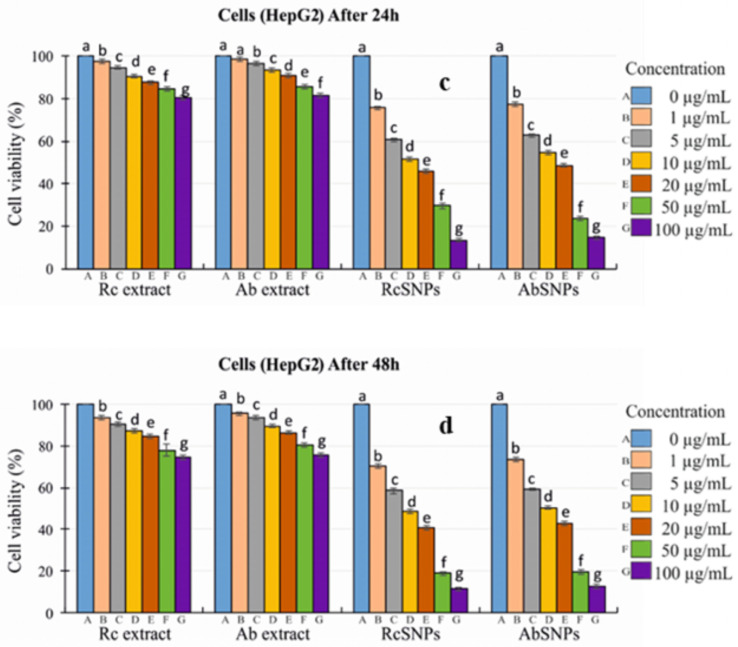
Cytotoxic activity of *Ricinus communis* (Rc) and *Aloe barbadensis* (Ab) extracts and their corresponding SNPs against B16F10 and HepG2 cell lines. Panels show (**a**) Rc and Ab treatments on B16F10 cells after 24 h, (**b**) B16F10 cells after 48 h, (**c**) HepG2 cells after 24 h, and (**d**) HepG2 cells after 48 h. Values with different superscripts (a–j) within the same variable are significantly different (*p* < 0.05).

Both RcSNPs and AbSNPs exhibited significant, dose-dependent cytotoxic effects against B16F10 and HepG2 cells (Figure 13a–d). The IC_50_ values at 24 h were 10 µg/mL for RcSNPs and 12 µg/mL for AbSNPs. Cytotoxicity increased further after 48 h of incubation, indicating a time-dependent enhancement of their anticancer activity. These results demonstrate the promising therapeutic potential of RcSNPs and AbSNPs as cytotoxic agents against melanoma and hepatocellular carcinoma cell lines.

## 4. Discussion

### 4.1. Synthesis and Optimization of Silver Nanoparticles (SNPs)

*Ricinus communis* and *Aloe barbadensis* plant extracts contain a variety of bioactive compounds, including alkaloids, glycosides, flavonoids, tannins, saponins, terpenoids, phenols, and reducing sugars, which can effectively reduce silver ions to silver nanoparticles (SNPs) and act as natural capping agents [38,76,77]. These phytochemicals highlight the medicinal potential of *R. communis* and *A. barbadensis*, as supported by previous studies [78,79]. In particular, the phenolic compounds exhibit redox properties that contribute to their antioxidant activity [80,81] and are largely responsible for the biological effects of the extracts. Consequently, both extracts are expected to demonstrate significant antioxidant, anti-inflammatory, and antibacterial activities. Literature indicates that phenolic and flavonoid compounds are more efficiently extracted in aqueous solutions and are biosynthesized via the phenylpropanoid pathway from phenylalanine [82]. The current findings align with total phenolic content (TPC) results reported for plant extracts from *Calophyllum tomentosum*, bearberry, olive leaves, *Euodia malayana*, *Gnetum gnemon*, *Haloxylon scoparium*, and *Khaya senegalensis* [7,83,84,85,86,87].

The color change of the reaction mixture serves as the initial indication of silver nanoparticle (SNP) formation. In this study, the solution color changed from yellowish to light brown and ultimately to dark brown (Figure 1), consistent with previous reports [88,89]. This color development is attributed to surface plasmon resonance (SPR), which arises from the collective oscillation of conduction electrons induced by an electromagnetic field interacting with electrons at the surface of metallic nanoparticles [90]. The SPR band’s position and width depend on the nanoparticle’s size and shape, the metal’s dielectric properties, and the surrounding environment [91]. UV–visible (UV–vis) spectroscopy, a simple and widely used technique, confirmed SNP synthesis, with SPR bands observed near 435 nm for *R. communis* and 442 nm for *A. barbadensis*, in agreement with the literature [92,93]. At higher silver nitrate concentrations, larger SNPs were formed, accompanied by broader SPR bands, likely due to the inhibitory effect of excess silver ions on nanoparticle formation, which slowed the reaction rate.

Another study reported that increasing the silver nitrate concentration enhanced silver nanoparticle (AgNP) synthesis, as evidenced by the intensified color of the reaction mixture due to the aggregation of silver ions, leading to greater nanoparticle formation [94]. Using 1 mM silver nitrate with *Solanum trilobatum* extract produced monodisperse AgNPs without aggregation. However, at 2–4 mM silver nitrate, a spectral band appeared at 460 nm, and at 5 mM, the SPR band shifted to 500 nm, indicating polydispersity and larger particle sizes. Increasing the silver nitrate concentration generally broadens the plasmon resonance band [95]. Similarly, increasing the leaf extract concentration to 4 mL (Figure 2b,g) resulted in a red shift of the SPR band and subtle changes in absorbance, reflecting changes in particle size [64]. Higher extract concentrations also increased absorption intensity. UV–vis spectroscopy is thus widely accepted for monitoring nanoparticles of controlled size and shape in aqueous suspensions. These observations are consistent with previous reports, where AgNP synthesis using *Pithophora dogonia* and *Cochlospermum religiosum* extracts exhibited SPR bands around 445 nm [96,97]. Collectively, these findings suggest that *R. communis* and *A. barbadensis* leaf extracts enable rapid and efficient synthesis of AgNPs, as confirmed by their UV–vis absorption spectra.

Similar observations were reported for silver nanoparticles (AgNPs) synthesized using *Malus domestica* [98], *Parthenium hysterophorus* [99], *Abies alba* and *Pinus sylvestris* [100]. Increasing the concentration of plant extract enhanced the absorption peak, indicating a greater reduction of silver ions and the formation of stable, well-defined AgNPs. At higher extract concentrations, phytochemicals not only act as reducing agents but also coat the nanoparticles, preventing agglomeration and improving stability [101].

The synthesis of silver nanoparticles (SNPs) was found to increase with reaction time; however, prolonged reaction periods can lead to instability due to agglomeration and increased particle size. Logeswari et al. reported SNP formation at 24–48 h at 37 °C using 1 mM silver nitrate and ammonium solution with ethanolic extracts of *Centella asiatica*, *Syzygium cumini*, *Citrus sinensis*, and *Solanum trilobatum*. The reaction mixture color changed from yellow to dark brown, and the resulting SNPs were irregular in shape with sizes ranging from 41 to 53 nm [71]. In contrast, SNPs synthesized from *Ocimum sanctum* began forming within 3 min, reaching optimal levels at 30 min. Absorption spectra recorded at 60, 90, and 120 min showed no significant differences, although the absorption peak bandwidth increased with nanoparticle polydispersity [102].

pH also plays a critical role in SNP synthesis. Lower pH values favor the formation of larger particles due to aggregation, whereas higher pH values promote nucleation and produce smaller, well-dispersed SNPs. For example, *Garcinia mangostana* extract produced large aggregates at pH 4, while highly dispersed small SNPs were obtained at pH 8. Neutral pH was found to be optimal for SNP formation [88]. Supporting this, Iravani Zolfaghari investigated SNP synthesis using pine bark extract with varying silver nitrate concentrations and phosphate buffers across a pH range of 3–11, confirming the significant influence of pH on nanoparticle size and dispersion [103].

The effect of pH on SNP synthesis has been widely reported. Using *Acalypha indica* extract, no SNP formation was observed under acidic conditions, whereas rapid color change and SNP formation occurred under alkaline conditions, with an absorption peak shifting to 500 nm; however, at pH 13, immediate aggregation was observed. At neutral pH, the reaction commenced upon addition of silver nitrate, with SNP formation completed within 30 min of incubation [104]. Similarly, when *Crataegus douglasii* fruit extract was used, the absorption wavelength decreased from pH 2–6 over 24 h. No SNPs were formed at pH 2 due to aggregation, while alkaline conditions significantly reduced aggregation [105]. For *Solanum trilobatum* extract, pH 5.8 inhibited nanoparticle synthesis due to limited accessibility of functional groups. At pH 8.8, a broad absorption band appeared at 480 nm, whereas at pH 7.8, a narrow peak at 440 nm corresponded to maximum SNP production [106].

Ndikau et al. synthesized spherical and stable silver nanoparticles (SNPs) using 0.001 M *Citrullus lanatus* extract and 250 g/L silver nitrate at 80 °C and pH 10 in a 4:5 ratio, obtaining nanoparticles with a diameter of 17.96 ± 0.16 nm [107]. Kumar et al. reported SNP synthesis at room temperature using 10% *Annona squamosa* extract and 1 mM silver nitrate for 4 h, producing spherical to irregular-shaped nanoparticles with an average size of 35 ± 5 nm [108]. Similarly, Das et al. prepared SNPs from *Sesbania grandiflora* leaves with 1 mM silver nitrate by incubating in the dark at 37 °C; the resulting spherical nanoparticles (10–25 nm) remained stable at room temperature for six months [109].

The effect of temperature on SNP synthesis has been extensively studied. Using *Solanum trilobatum*, no SPR band was observed at 20 °C, whereas distinct SPR peaks appeared at 35, 45, and 70 °C, with the highest yield achieved at the highest temperature. The SPR peak shifted from 460 nm to 440 nm (blue shift), indicating that higher temperatures strongly influence nanoparticle properties [110]. Generally, increasing the temperature (25–150 °C) enhances SNP production, intensifies absorbance, and reduces nanoparticle size, resulting in a more pronounced SPR band [14]. Similarly, SNP synthesis from *Garcinia mangostana* leaf extract between 37 and 90 °C showed increased nanoparticle yield with rising temperature. Initially, particle size decreased due to reduced aggregation, but above 75 °C, crystal formation around the nuclei caused a decline in absorbance [111].

In the present study, the optimized conditions for *R. communis* silver nanoparticles (RcSNPs) synthesis were 1 mM AgNO_3_, 2 mL leaf extract, 25 °C, pH 11, and a 5 min reaction time. For *A. barbadensis* silver nanoparticles (AbSNPs), the optimal conditions were 2 mM AgNO_3_, 1 mL leaf extract, 25 °C, pH 12, and a 3 min reaction time.

### 4.2. Proposed Mechanism for RcSNPs and AbSNPs Synthesis

In this study, *R. communis* and *A. barbadensis* aqueous leaf extracts were used for the plant-mediated synthesis of silver nanoparticles (SNPs). The process is initiated by the phenolic compounds and flavonoids present in the extracts. Phytochemical analysis of *R. communis* extract revealed the presence of alkaloids, phenolic compounds, flavonoids, sugars, and proteins. Among these, phenolic compounds and flavonoids act as efficient reducing agents, whereas proteins and certain other phytochemicals serve as capping and stabilizing agents for SNPs [112]. The enol groups of phenolic compounds and flavonoids can release electrons through O–H bond cleavage, and these electrons reduce Ag^+^ to Ag^0^. Additionally, proteins in *R. communis* extract are expected to stabilize the nanoparticles by capping them [113]. FTIR analysis supports this mechanism, indicating the involvement of hydroxyl and amide groups in the reduction and capping of RcSNPs.

Previous studies indicate that metallic ion reduction can occur via oxidation of amino or hydroxyl groups [114], or through specific terpenoids and alkaloids present in *A. barbadensis* extract. In this case, hydroxyl groups in alkaloids, glycosides, and steroids transfer electrons to silver ions, facilitating SNP formation [115]. Reduction of metallic ions is often achieved through oxidation of O–H groups to carbonyl groups [116]. The resulting nanoparticles may bind to proteins released from the *A. barbadensis* extract, producing protein-capped SNPs.

Stability studies confirmed that SNPs synthesized using these plant extracts are more stable than those produced via conventional chemical methods. These findings are consistent with reports from other researchers, highlighting the effectiveness of plant-mediated synthesis in producing stable, protein-capped nanoparticles.

### 4.3. Characterization of SNPs

Dynamic light scattering (DLS) was employed to determine the particle size distribution of the synthesized nanoparticles, providing information on the hydrodynamic diameter, size distribution, and polydispersity index (PDI). The measured negative zeta potential values indicate that the nanoparticles are highly stable, and dispersity analysis confirmed that they are well distributed. These findings are consistent with previously reported values [117] and correlate with UV–vis spectroscopy results. According to Gengan et al., a zeta potential greater than +30 mV or less than –30 mV reflects high colloidal stability [118]. Negative values typically arise from the incorporation of bioactive molecules from the plant extract [119], which induce strong electrostatic repulsion between nanoparticles and prevent aggregation [120]. The observed strong negative zeta potential may result from the shielding effect of biologically active plant compounds [121].

Biogenic SNPs are further stabilized through capping by phenolic compounds present in the leaf extracts, which also facilitate the reduction of Ag^+^ ions. To identify the phenolic constituents involved in capping, FTIR analysis was performed on RcSNPs, AbSNPs, and their respective leaf extracts. The analysis confirmed the presence of plant-derived phenolic functional groups on the nanoparticle surface, contributing to the excellent stability profile of the prepared SNPs [122,123].

The morphology and size distribution of silver nanoparticles (SNPs) are critical determinants of their physicochemical properties and biological activities [124]. In this study, SEM revealed that the synthesized SNPs exhibited diverse morphologies, including spherical, cubic, triangular, and rectangular forms. While the particles were relatively well distributed, some agglomeration was observed, likely due to limited stabilization from weak capping agents. TEM analysis further confirmed that the majority of nanoparticles were spherical, consistent with UV–vis and SEM observations, and indicated smaller particle sizes (15–20 nm for RcSNPs and 23–28 nm for AbSNPs).

Recent studies have emphasized the importance of nanoparticle size and shape in biological applications. Nanoparticles within the 1–100 nm range, such as those obtained here, provide a high surface area to volume ratio, which enhances interactions with microbial cells and biological targets [125]. Spherical nanoparticles, in particular, exhibit lower surface energy and higher thermodynamic stability, contributing to uniform dispersion and consistent bioactivity [126]. The presence of triangular and cubic morphologies may further enhance antimicrobial and antioxidant activities due to higher surface energy and increased reactive sites [127].

Agglomeration, as observed in some particles, is a common challenge in green nanoparticle synthesis. It can reduce the effective surface area and potentially decrease biological efficacy [128]. Factors influencing agglomeration include the concentration and type of capping agents, pH, and ionic strength of the synthesis medium. Optimizing these parameters can enhance particle stability and maintain the desirable nanoscale features for biomedical applications [129].

Overall, the SEM and TEM analyses indicate that green synthesis using *Ricinus communis* and *Aloe barbadensis* leaf extracts produces predominantly spherical, uniformly sized SNPs with minor agglomeration. The diversity in shapes and nanoscale dimensions suggests a potential for enhanced biological activity, supporting their use in antimicrobial, antioxidant, anti-inflammatory, and anticancer applications [130]. Future work may focus on fine-tuning synthesis conditions to minimize agglomeration and improve nanoparticle stability.

Thermogravimetric analysis (TGA) and differential scanning calorimetry (DSC) were employed to assess the thermal stability and compositional characteristics of the silver nanoparticles (SNPs) synthesized using *R. communis* and *A. barbadensis* leaf extracts. TGA revealed an initial weight loss around 90 °C, indicative of the desorption of surface-bound biological compounds, such as proteins and polyphenols, which play a crucial role in the reduction and stabilization of silver ions during nanoparticle synthesis [131]. The DSC analysis demonstrated a single-stage endothermic peak corresponding to the decomposition of these organic stabilizers, aligning with the TGA results. This congruence suggests that the phytochemicals not only reduce Ag^+^ to Ag^0^ but also contribute to the thermal stability of the nanoparticles by forming a protective layer around them [132,133].

Collectively, the TGA and DSC analyses indicate that the synthesized SNPs possess good thermal stability up to approximately 90 °C, which is advantageous for their potential applications in various biomedical and industrial fields [134]. The presence of plant-derived stabilizing agents further underscores the eco-friendly nature of the green synthesis method employed.

### 4.4. Therapeutic Evaluation of SNPs

The antimicrobial activity of SNPs was evaluated against various pathogenic strains. The diameter of inhibition zones increased with higher SNP concentrations, indicating a dose-dependent effect [135]. Smaller nanoparticles, with their larger surface area, exhibited enhanced bactericidal activity [136]. The proposed mechanisms underlying the antimicrobial properties of plant-synthesized SNPs include (i) interaction of silver ions with thiol-containing proteins, leading to protein denaturation and loss of function; (ii) binding to negatively charged DNA, causing structural damage and inhibiting replication; (iii) inhibition of respiratory enzymes (e.g., hydrogenase II) and cofactors (e.g., NADH), disrupting cellular respiration; (iv) induction of reactive oxygen species (ROS) such as hydroxyl radicals and superoxide anions, interfering with the cell respiration cycle; and (v) direct penetration of silver ions into microbial cell walls, resulting in cell death [137,138,139]. Optimized RcSNPs and AbSNPs exhibited superior antimicrobial activity compared with previously reported *R. communis* and *A. barbadensis*-derived nanoparticles, attributed to their smaller size, improved stability, and spherical morphology [140,141,142,143,144].

The anti-inflammatory activity of SNPs was evaluated using the protein denaturation assay. Leaf extract–encapsulated SNPs significantly inhibited bovine serum albumin (BSA) denaturation, even at lower concentrations than standard anti-inflammatory agents. This effect is likely due to the synergistic action of SNPs and phytochemicals in *R. communis* and *A. barbadensis*, which also exhibited substantial antiprotease activity [145,146,147,148]. Protein denaturation is a well-established cause of inflammation, as it results in loss of biological activity and, in some arthritic conditions, the production of autoantigens. Denaturation involves alterations in hydrogen bonding, disulfide linkages, electrostatic interactions, and hydrophobicity [149].

The antioxidant potential of SNPs was evaluated using DPPH and H_2_O_2_ scavenging assays. Free radicals contribute to numerous human diseases, including arthritis, atherosclerosis, reperfusion injury, and cancer [150,151]. At higher concentrations, SNPs effectively scavenged DPPH radicals by electron donation, while also demonstrating greater reducing power than ascorbic acid. In the presence of H_2_O_2_, SNPs promoted ROS generation, including hydroxyl radicals, which could induce oxidative stress through mitochondrial pathways, cathepsin leakage, and potassium efflux [152,153,154,155]. These results align with prior reports on H_2_O_2_ scavenging by *Abutilon indicum* leaf extract [156].

SNPs also exhibited notable anti-tyrosinase activity. Tyrosinase, a copper-containing metalloenzyme, catalyzes the oxidation of tyrosine to dihydroxyphenylalanine (DOPA) and subsequently to DOPA quinone, which are key intermediates in melanin biosynthesis [157,158]. Overactivity of tyrosinase can lead to hyperpigmentation disorders, including melasma, freckles, and age spots. Biogenic RcSNPs and AbSNPs demonstrated significant inhibition of tyrosinase activity, which is likely attributed to the functional groups from plant phytochemicals capping the nanoparticle surfaces [159]. These capping molecules may interact with the copper active site of tyrosinase or with the substrate binding site, thereby preventing enzymatic oxidation of tyrosine. This dual mechanism—physical adsorption of the enzyme onto the nanoparticle surface and chemical interaction via bioactive functional groups-enhances the potential of these SNPs as natural skin-lightening or depigmenting agents in cosmetic and therapeutic applications [160].

The cytotoxicity and anticancer potential of RcSNPs and AbSNPs were assessed using the MTT assay, which demonstrated a significant reduction in cell viability in a dose-dependent manner. The SNPs induced apoptosis via mitochondrial membrane disruption and ROS production, activating the caspase-9/3 signaling pathway [161,162,163]. The release of cytochrome c from the mitochondrial intermembrane space further contributed to mitochondrial stress, while apoptosis-regulating genes Bcl-2 and Bax mediated the loss of mitochondrial integrity [164].

Compared with previously reported in vitro studies on *R. communis* and *A. barbadensis*, the cytotoxic effects observed in this study were more pronounced, indicating that these biogenic SNPs are promising candidates for anticancer applications [38,140,144,145,165,166]. The incorporation of phytochemicals during green synthesis likely enhances the nanoparticles’ stability and bioactivity, contributing to their improved therapeutic profile. To further illustrate the novelty and comparative advantages of the present RcSNPs and AbSNPs, Table 4 summarizes key physicochemical and biological parameters of similar plant-derived AgNPs reported in recent literature.

As summarized in Table 4, the nanoparticles synthesized in this work exhibit smaller and more uniform sizes, faster formation rates, and higher antimicrobial and cytotoxic efficacy than most previously reported green-synthesized AgNPs, underscoring the efficiency of *R. communis* and *A. barbadensis* extracts as dual reducing and stabilizing agents.

Overall, the study demonstrates that the phytochemical components of *R. communis* and *A. barbadensis* enable effective synthesis of stable, bioactive AgNPs with multifunctional properties. These findings align with recent advances in phytogenic nanoparticle research and highlight their potential in biomedical applications

## 5. Conclusions

This study demonstrates the efficient green synthesis of silver nanoparticles using *Ricinus communis* and *Aloe barbadensis* leaf extracts as reducing and capping agents. Optimized conditions yielded uniformly sized, spherical nanoparticles with enhanced stability. RcSNPs and AbSNPs exhibited potent antibacterial, antifungal, anti-inflammatory, antioxidant, anticoagulant, anti-tyrosinase, and cytotoxic activities, surpassing the efficacy of plant extracts and comparable reports. These findings position phytogenic SNPs as promising candidates for multifunctional biomedical and biopharmaceutical applications, offering broad therapeutic efficacy with reduced toxicity. Future work should validate their in vivo performance and explore integration into advanced drug delivery systems.

## Figures and Tables

**Table 1:** Phytochemical analysis of *Ricinus communis* and *Aloe barbadensis* leaf extracts.

Phytochemicals	Aqueous Extract		Methanolic Extract	
	*Ricinus communis*	*Aloe barbadensis*	*Ricinus communis*	*Aloe barbadensis*
Alkaloids	−	+	+	+
Steroids	−	−	+	+
Flavonoids	+	+	+	+
Terpenoids	+	−	+	+
Glycosides	+	−	−	−
Phenols	−	+	+	+
Tannins	+	+	+	−
Saponins	+	−	+	+
Reducing sugars	+	+	+	+

**Table 2:** Zones of inhibition (mm) of RcSNPs, AbSNPs, standard antimicrobial drugs, and leaf extracts of *Ricinus communis* (Rc) and *Aloe barbadensis* (Ab) against pathogenic bacterial and fungal strains.

Microorganisms		RcSNPs		AbSNPs		Standard		Control	
		50 μL	100 μL	50 μL	100 μL	Gentamycin	Fluconazole	Rc Extract	Ab Extract
**Bacteria**	*S. aureus*	18.32 ± 0.3	20.2 ± 0.2	17.2 ± 0.1	20.3 ± 0.1	25.2 ± 0.3	-	12.3 ± 0.4	10.4 ± 0.5
	*S. typhi*	18.1 ± 0.7	19.3 ± 0.5	14.5 ± 0.1	18.6 ± 0.1	27.8 ± 0.1	-	14.6 ± 0.4	8.6 ± 0.6
	*E. coli*	17.6 ± 0.9	18.4 ± 0.7	16.1 ± 0.05	17.3 ± 0.05	23.4 ± 0.1	-	11.2 ± 0.7	11.7 ± 0.3
**Fungi**	*C. albicans*	20.1 ± 0.7	22.3 ± 0.8	17.25 ± 0.2	20.5 ± 0.1	-	22.8 ± 0.7	10.7 ± 0.9	13.6 ± 0.2
	*A. niger*	19.4 ± 0.7	21.4 ± 0.7	22.5 ± 0.3	24.3 ± 0.4	-	23.21 ± 0.7	9.8 ± 0.2	17.1 ± 0.8

50 µg/mL, 50–100 µL per well. Experiments were performed in triplicates and the values are expressed as mean+ SD.

**Table 3:** Tyrosinase inhibitory activity (%) of silver nanoparticles synthesized from Ricinus communis (RcSNPs) and Aloe barbadensis (AbSNPs).

Testing Material	Tyrosinase Inhibition (%)
RcSNPs	97.98 ± 1.5
AbSNPs	98.43 ± 1.5
Ascorbic acid	99.23± 0.1

Values represent mean ± SD (*n* = 3).

**Table 4:** Comparative summary of phytogenic silver nanoparticles reported in recent literature versus the present study.

Source (Plant Extract)	Particle Size (nm)	Shape	Synthesis Features	Antimicrobial Activity (Zone of Inhibition, mm)	Cytotoxic/Other Bioactivity	Reference
*Azadirachta indica* (Neem)	25–45	Spherical	Moderate stability; slow reduction (4 h)	*E. coli*—17 ± 0.5 mm	Moderate anticancer (IC_50_ > 100 µg/mL)	[167]
*Camellia sinensis* (Green tea)	30–60	Quasi-spherical	Good dispersion; requires heating	*S. aureus*—18 ± 0.7 mm	Low cytotoxicity (IC_50_ > 150 µg/mL)	[168]
*Moringa oleifera*	20–40	Spherical	High yield; moderate stability	*E. coli*—19 ± 0.4 mm	Limited antiproliferative effect	[169]
*Ocimum sanctum* (Tulsi)	18–30	Irregular	Requires alkaline pH > 9	*A. baumannii*—15 ± 0.8 mm	Moderate anticancer (IC_50_ > 125 µg/mL	[170]
*Ricinus communis* (present study)	16–22	Uniform spherical	Rapid synthesis (≤30 min); high stability (>3 mo)	*S. aureus*—20.2 ± 0.9 mm; *C. albicans*—22.3 ± 0.8 mm	Strong cytotoxicity (B16F10 IC_50_ ≈ 52 µg/mL)	This study
*Aloe barbadensis* (present study)	18–24	Spherical	Good colloidal stability; reproducible synthesis	*A. niger*—21.8 ± 0.4 mm; *E. coli*—17.3 ± 0.05 mm	Significant cytotoxicity (HepG2 IC_50_ ≈ 60 µg/mL)	This study

## Data Availability

The original data supporting the findings of this study are included in the article. Further inquiries can be directed to the corresponding author.
